# Tongue color parameters in predicting the degree of coronary stenosis: a retrospective cohort study of 282 patients with coronary angiography

**DOI:** 10.3389/fcvm.2024.1436278

**Published:** 2024-08-30

**Authors:** Jieyun Li, Danqun Xiong, Leixin Hong, Jiekee Lim, Xiangdong Xu, Xinang Xiao, Rui Guo, Zhaoxia Xu

**Affiliations:** ^1^ School of Traditional Chinese Medicine, Shanghai University of Traditional Chinese Medicine, Shanghai, China; ^2^ Shanghai key Laboratory of Health Identification and Evaluation, Shanghai, China; ^3^ Department of Cardiology, Jiading District Central Hospital, Shanghai, China

**Keywords:** tongue analysis, coronary angiography, coronary artery stenosis, retrospective cohort study, machine learning

## Abstract

**Purpose:**

This retrospective cohort study aimed to analyze the relationship between tongue color and coronary artery stenosis severity in 282 patients after underwent coronary angiography.

**Methods:**

A retrospective cohort study was conducted to collect data from patients who underwent coronary angiography in the Department of Cardiology, Shanghai Jiading District Central Hospital from October 1, 2023 to January 15, 2024. All patients were divided into four various stenosis groups. The tongue images of each patient was normalized captured, tongue body (TC_) and tongue coating (CC_) data were converted into RGB and HSV model parameters using SMX System 2.0. Four supervised machine learning classifiers were used to establish a coronary artery stenosis grading prediction model, including random forest (RF), logistic regression, and support vector machine (SVM). Accuracy, precision, recall, and F1 score were used as classification indicators to evaluate the training and validation performance of the model. SHAP values were furthermore used to explore the impacts of features.

**Results:**

This study finally included 282 patients, including 164 males (58.16%) and 118 females (41.84%). 69 patients without stenosis, 70 patients with mild stenosis, 65 patients with moderate stenosis, and 78 patients with severe stenosis. Significant differences of tongue parameters were observed in the four groups [TC_R (*P *= 0.000), TC_G (*P *= 0.003), TC_H (*P *= 0.001) and TC_S (*P *= 0.024),CC_R (*P *= 0.006), CC_B (*P *= 0.023) and CC_S (*P *= 0.001)]. The SVM model had the highest predictive ability, with AUC values above 0.9 in different stenosis groups, and was particularly good at identifying mild and severe stenosis (AUC = 0.98). SHAP value showed that high values of TC_RIGHT_R, low values of CC_LEFT_R were the most impact factors to predict no coronary stenosis; high CC_LEFT_R and low TC_ROOT_H for mild coronary stenosis; low TC_ROOT_R and CC_ROOT_B for moderate coronary stenosis; high CC_RIGHT_G and low TC_ROOT_H for severe coronary stenosis.

**Conclusion:**

Tongue color parameters can provide a reference for predicting the degree of coronary artery stenosis. The study provides insights into the potential application of tongue color parameters in predicting coronary artery stenosis severity. Future research can expand on tongue features, optimize prediction models, and explore applications in other cardiovascular diseases.

## 1 Introduction

Coronary angiography is an important diagnostic tool. It can reveal the internal structure of the coronary artery by injecting a contrast agent and using x-ray technology, to accurately judge the degree of coronary artery obstruction. It has an irreplaceable position in the field of cardiovascular medicine (1). The degree of coronary artery occlusion is directly related to the heart's blood supply, which is the key basis for evaluating the risk of coronary heart disease and selecting a treatment plan. Coronary heart disease (CHD) is a common cardiovascular disease, its occurrence and development are closely related to the degree of coronary artery stenosis. Coronary angiography is an important reference index for diagnosing and treating CHD (2).

Tongue diagnosis, as one of the unique methods of traditional Chinese medicine (TCM) diagnosis, has always been regarded as an important way to evaluate health status and identify diseases. As a crucial component of the TCM diagnostic system, it provides insights into the body's internal pathological changes. Moreover, it is simple, convenient, and doesn't necessitate complex procedures or expensive equipment.The tongue image includes the tongue body, which refers to the muscle and vein tissue of the tongue; the tongue coating, which refers to a layer of moss attached to the surface of the tongue, is mainly composed of food debris, bacteria and saliva (3). Tongue examination involves checking the color, shape, thickness, and dryness of the tongue and tongue coating (4). Advancements in objective tongue diagnosis technology have further enhanced its objectivity and accuracy, making it even more valuable in clinical practice and providing a more reliable reference for disease diagnosis and treatment (3). Modern medical research showed that the blood supply of the tongue mainly comes from the branch of the external carotid artery—the tongue artery (5, 6). When coronary artery atherosclerosis leads to myocardial ischemia, the pumping function of the heart is damaged, which will affect the blood supply of the tongue and lead to changes in the tongue image (7, 8). In the study of cardiovascular diseases, tongue image changes are closely related to heart function, blood circulation and disease progression (9). Therefore, we hypothesized that the tongue (tongue body color and tongue coating color) can provide direct clues to the pathologic state of the heart (such as the degree of coronary artery stenosis) to a certain extent.

**Figure 1:**
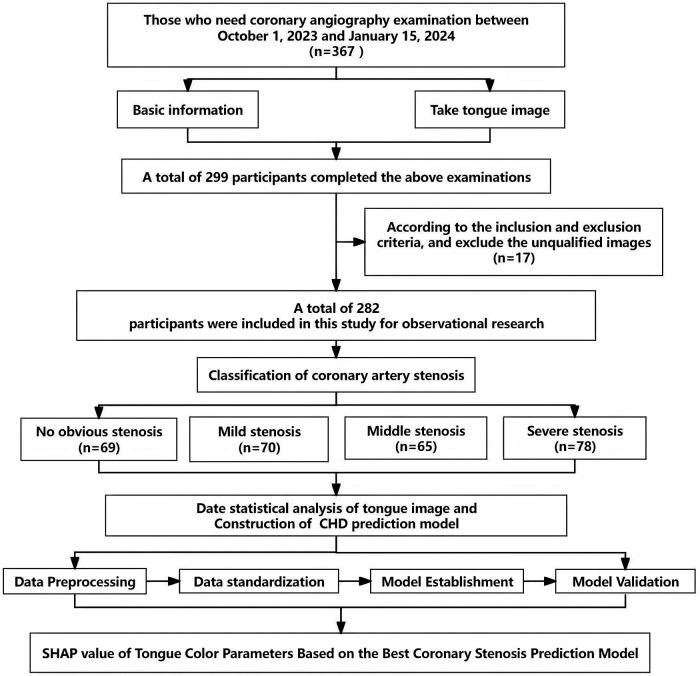
Flow chat.

By leveraging advanced image processing techniques based on RGB and HSV color models, the color parameters of both tongue body and coating can be extracted, enabling observation of the correlation between changes in tongue image color and the degree of coronary artery stenosis. Therefore, the purpose of this study was to analyze the relationship between tongue color and coronary angiography results in 282 patients undergoing coronary angiography by retrospective cohort study. According to the results of coronary angiography, these patients were divided into different groups with different degrees of coronary stenosis (no stenosis, mild, moderate and severe). The camera was used to obtain pictures of the patient's tongue and extract the color parameters of the tongue body and coating. These data were then used to train a machine learning model to analyze the relationship between tongue color changes and the degree of coronary artery stenosis, and to evaluate the diagnostic performance of the model through the ROC curve (receiver operator characteristic curve), SHAP value (SHapley Additive Explanatory Values) and other methods, so as to verify the practical value of tongue color in judging the degree of coronary artery stenosis. The detailed flow chart of this study is shown in Figure 1.

## 2 Material and methods

### 2.1 Study population

This study was a retrospective cohort study, and data were collected from patients who underwent coronary angiography in the Department of Cardiology, Jiading District Central Hospital, Shanghai, China, between October 1, 2023 and January 15, 2024. During this period, all patients who underwent angiography were selected, and their demographic, clinical characteristics, angiographic results, and surgery-related characteristics were collected. Strengthening the reporting of observational studies in Epidemiology (STROBE) checklist helped guide the action and reporting review of this cohort study (10). Transparent reporting of a multivariable prediction model for individual prognosis or diagnosis (TRIPOD) statement checklist was used to guide the establishment and validation of clinical prediction models for coronary stenosis based on tongue color parameters (11). The study protocol was approved by the Institutional Review Board of Shanghai University of Traditional Chinese Medicine (No. 2023-3-10-08-07) and was implemented in accordance with relevant guidelines and regulations.

### 2.2 Inclusion and exclusion criteria

Inclusion criteria: (1) patients who underwent coronary angiography during the study period; (2) patients with complete clinical, tongue parameter, and physical and chemical index data.

Exclusion criteria: (1) patients with severe heart disease (e.g., valvular heart disease, congenital heart disease); (2) patients with malignant tumors or other serious systemic diseases; (3) patients unwilling to participate in the study or having incomplete data.

Additionally, following the 2018 ESC/EACTS Guidelines for Myocardial Revascularization (12), and the 2021 ACC/AHA/SCAI Guideline for Coronary Artery Revascularization (13), the degree of coronary artery stenosis was employed as the primary evaluation index. The degree of stenosis is typically expressed as a percentage representing the proportion of stenosis to normal vascular diameter. Based on this degree, enrolled patients were categorized into four groups: no obvious stenosis group, mild stenosis group (<50%), moderate stenosis group (50%–74%), and severe stenosis group (≥75%).

### 2.3 Data acquisition and extraction

#### 2.3.1 Clinical index collection

Data were collected from 282 patients undergoing coronary angiography. Basic information such as the patient's age, gender, height, weight, blood pressure and heart rate, past medical and surgical history, as well as coronary angiography results were included. Coronary angiography is performed by professional cardiologists in accordance with standard operating procedures to ensure reliable and accurate results and is filled in a unified clinical investigation record sheet to provide a solid basis for subsequent data analysis.

#### 2.3.2 Tongue image parameters collection

Tongue images were captured using a Canon PowerShot SX720 HS digital camera and a color card. The participants refrained from drinking water for 30 min and abstained from eating for one hour. After a period of rest lasting at least 5 min, they were instructed to open their mouths and extend their tongues while sitting or lying down, relaxing their facial muscles and maintaining the position for 3 s in order to obtain tongue images. The collection of tongue diagnosis parameters was carried out by four experienced traditional Chinese medicine practitioners (JY-L, LX-H, XA-X and R-G), with the data being collected within 6 h before coronary angiography.

#### 2.3.3 Tongue image parameters extraction

**Figure 2:**
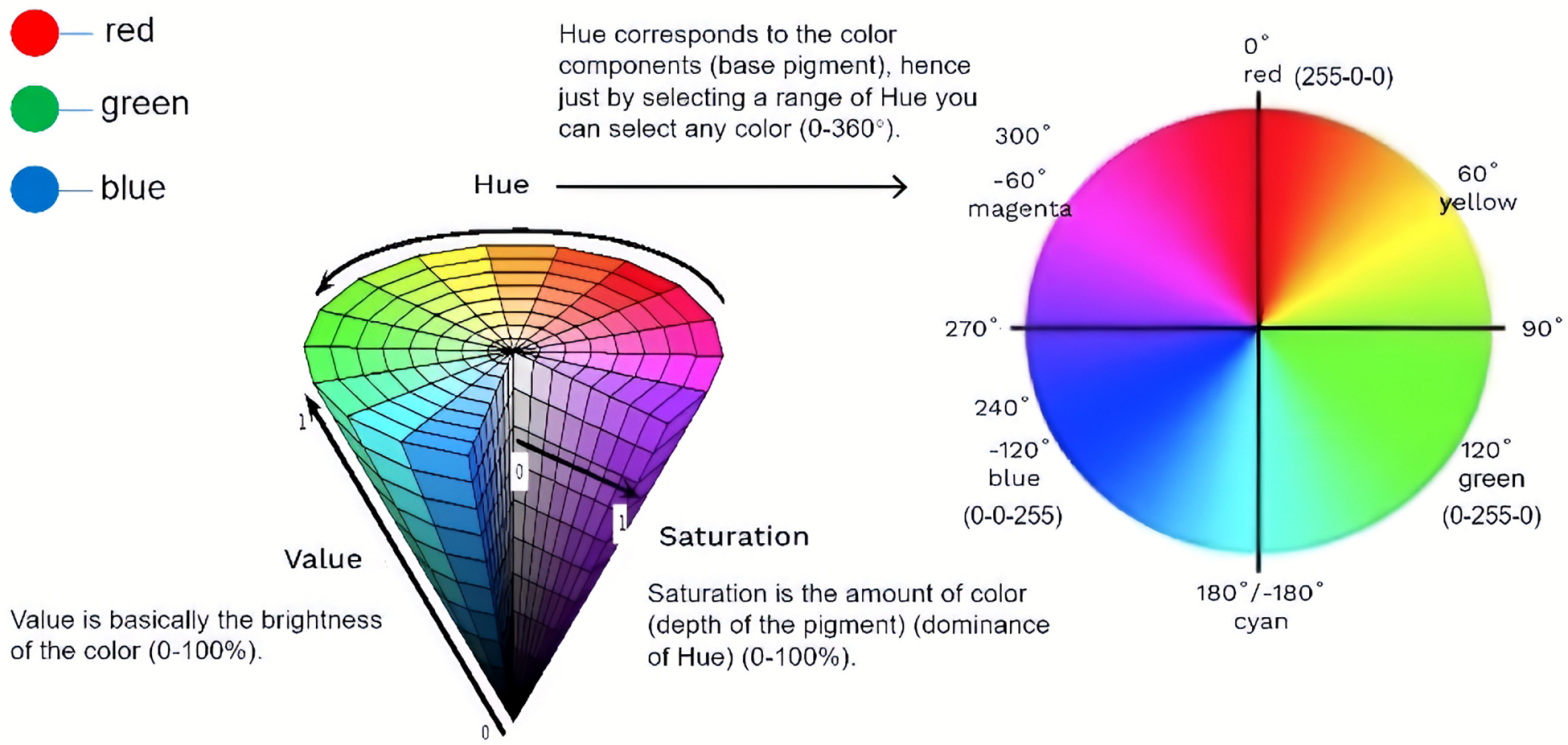
RGB and HSV color model. This diagram depicts and illustrates the relationship between the RGB and HSV color models. It shows how the RGB color space can be transformed into the HSV color space, allowing for easier manipulation of colors based on hue, saturation, and value.

The tongue tissue and tongue coating were separated and segmented using the “SMX System 2.0 tongue image analysis software” (Registration No. 2008SR12316). The color parameters of the tongue tissue and tongue coating were described using the RGB model and HSV model as measurement indexes. As depicted in Figure 2, the RGB model is an additive color model that represents various colors through different combinations of intensity for three primary colors: red, green, and blue. Each color's intensity is typically represented by an integer ranging from 0 to 255, where 0 signifies the absence of the color and 255 denotes its maximum intensity (14). On the other hand, the HSV model is a color model based on human visual perception. In this particular model, H value (hue) represents fundamental attributes such as red, green, blue, etc.; S value (saturation) indicates purity or brightness; V (value) signifies lightness (15).

**Figure 3:**
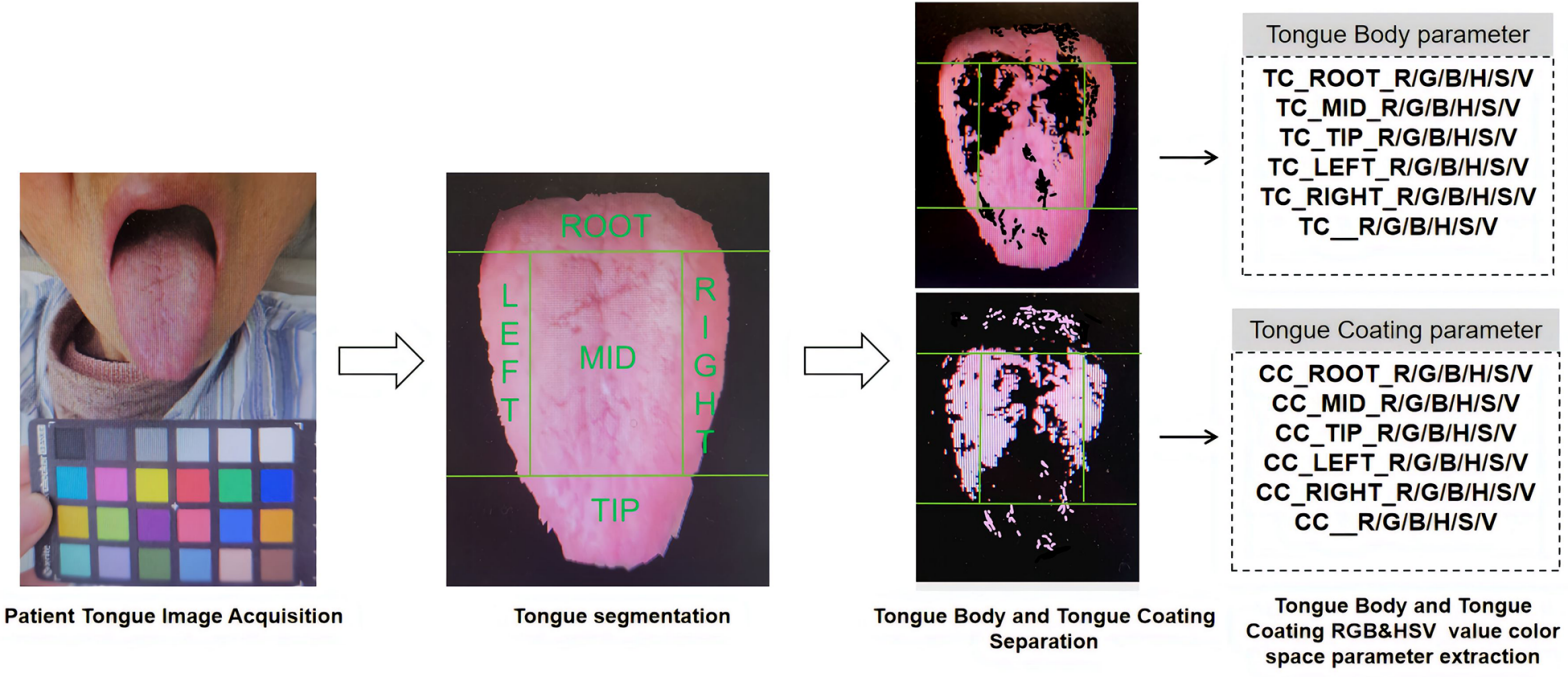
Overview of the tongue image parameters collection and extraction approach.

In addition, as the tongue is a complex organ with its surface covered with papillae and taste buds, it may show specific tongue features in different regions of the tongue under the influence of different diseases or pathological processes. As shown in Figure 3, in order to more systematically observe and analyze these features, the system parameters divide the tongue into root, middle, tip, left and right parts.

### 2.4 Feature selection

Feature selection is highly critical in the clinical prediction model establishment process, as some features may have no effect on the prediction results or even interfere with the effectiveness of the prediction model. While machine learning-based feature selection methods possess the capability to automatically select features, their outcomes are intricate and challenging to interpret. Conversely, non-parametric tests typically yield more intuitive and interpretable results (16). By comparing differences in tongue chart analysis parameters among different groups, we can directly comprehend which features are associated with the degree of coronary artery stenosis. Furthermore, non-parametric tests do not rely on specific data distribution assumptions and apply to various types of data distributions. Moreover, non-parametric tests exhibit robustness and provide reliable statistical results even when dealing with small sample sizes and outliers (17). This holds immense significance for exploring the relationship between tongue chart parameters and the degree of coronary artery stenosis with limited sample sizes.

### 2.5 Machine learning classification

Machine learning algorithms can extract useful information from large amounts of data and make objective and accurate predictions through algorithm models. We hope to more comprehensively evaluate the application value of tongue diagnosis parameters in predicting different degrees of coronary artery stenosis by analyzing tongue parameters through machine learning. In this study, we explored and validated the hypothesis that tongue color parameter analysis can be used as a clinical auxiliary diagnostic method by establishing a coronary artery stenosis grading prediction model based on tongue color parameters. We used four supervised machine learning classifiers, random forest (RF) (18), logistic regression (LR) (19), XGBoost (20) and support vector machine (SVM) (21). These are classical machine learning models for building prediction models, and these prediction models have been proven effective in previous studies on heart disease (22–24). All the machine learning algorithms mentioned above were written using Python 3.9 and Scikit-Learn library.

In each modelling session, the dataset is randomly divided into a training set and a test set in a 7:3 ratio. The training set is used for model training, and the test set is used to verify the model performance. All eigenvalues are normalized by a Standard Scaler, which works by subtracting the mean and dividing by the variance, so that all values are centred at zero and the variance is 1. Layered 10-fold cross-validation and grid search are used to optimize parameters during model training. Classification metrics used to evaluate model training and validation performance are accuracy, precision, recall, and F1 score (25). Accuracy is the most common classification metric. It represents the proportion of the total number of samples that the model correctly predicts. Precision represents the proportion of samples that are actually positive among all samples that the model predicts as positive. This helps us understand the accuracy of the model in predicting positive classes. The recall represents the proportion of samples that the model correctly predicts as positive among all samples that are actually positive. The F1 score is the harmonic mean of precision and recall. It takes into account the performance of precision and recall. The higher the F1 score, the better the model performs in both precision and recall. The ROC curve is a graphical tool used to evaluate the performance of classification models, which shows the relationship between the true positive rate (TPR) and the false positive rate (FPR) at different classification thresholds. The AUC value (Area Under Curve) represents the area under the ROC curve, which reflects the overall performance of the model at different classification thresholds. The AUC value ranges from 0.5 to 1, where 0.5 indicates that the model performance is equivalent to random guessing, and 1 indicates that the model performance is perfect. The AUC values of the four models were compared to show the performance differences of tongue color feature parameters in the classification task of coronary artery stenosis degree (detailed calculations are shown in the Supplementary Material).

In order to enhance the model's credibility and facilitate its utilization by clinicians, it is imperative to not only report the prediction results but also interpret the model. Traditional feature importance can merely indicate the significance of feature values without explicitly describing their impact on prediction outcomes. Therefore, in our study, we employed SHapley Additive Explanatory Values (SHAP) for visualization analysis. SHAP values not only unveil feature importance but also provide a quantitative measure to assess each feature's contribution to prediction results. This enables us to accurately compare the influence of different tongue features on model output outcomes. Furthermore, SHAP values can reveal feature interactions, allowing us to gain a deeper understanding of how these features interact with one another and comprehensively comprehend the relationship between tongue color and coronary artery stenosis severity. Leveraging an optimal machine learning algorithm for grading coronary artery stenosis, this study will utilize the Python 3.9 version of the SHAP library's summary graph to plot SHAP values for each sample.

### 2.6 Statistical analysis

The statistics of basic information was used for data analysis using SPSS software. Count data were presented as numbers (%) and measurement data were expressed as mean ± standard deviation (SD) if they followed a normal distribution. In cases where the data did not follow a normal distribution or had uneven variance, median and interquartile range M (P25, P75) were used to represent the data (26). The Kruskal-Wallis H test was employed to compare differences between different tongue color characteristic parameters, with a significance level of *P *< 0.05 (27). Following the statistical analysis, radar charts were utilized to visually display variations in tongue color characteristic parameters among different coronary artery stenosis groups for both tongue and tongue coating.

## 3 Result

### 3.1 Basic information

After excluding 68 subjects who declined to participate in the study cohort and 17 subjects with missing data on tongue color parameters, a total of 282 subjects underwent coronary angiography. This included 164 males (58.16%) and 118 females (41.84%). As depicted in Table 1, the average age of the four patient groups ranged from 63 to 68 years old. Systolic blood pressure, diastolic blood pressure, and heart rate were all within normal ranges, with no statistically significant differences observed between the groups. Following professional clinical consultation, patients primarily reported symptoms such as chest tightness, chest pain, and palpitations. The most prevalent past medical history included hypertension, diabetes mellitus, coronary heart disease, and cerebral infarction. Among patients undergoing coronary angiography, three-vessel disease was the most common condition observed with a higher proportion of severe stenosis cases (15.96%), followed by single-vessel disease where mild stenosis accounted for the largest proportion (13.48%). In all four patient groups examined in this study cohort, the left anterior descending branch was found to be the most frequently affected vessel followed by the right coronary artery branch and left circumflex branch; particularly notable among patients with severe stenosis. Patients with different degrees of stenosis had statistically significant differences in their complaints of chest stuffiness, history of hypertension and CHD, as well as the left anterior descending artery (*P* < 0.05).

**Table 1:** Basic characteristic information of patients.

Factor	Classification of stenosis *N* (%)/(Mean ± SD)/M (P25, P75)				*P*
	No stenosis (*n* = 69)	Mild stenosis (*n* = 70)	Moderate stenosis (*n* = 65)	Severe stenosis (*n* = 78)	
Age (year)	66.28 ± 11.69	63.90 ± 10.83	68.02 ± 10.77	68.01 ± 9.57	0.104
Sex (F/M)	39 (13.83)/30 (10.64)	37 (13.12)/33 (11.70)	19 (6.71)/46 (16.31)	23 (8.16)/55 (19.50)	/
Height (cm)	163.07 ± 7.96	163.50 ± 8.85	166.05 ± 7.65	165.19 ± 7.07	0.11
Weight (kg)	66.000 (58.5, 74.0)	65.500 (58.6, 75.2)	70.000 (60.0, 80.0)	68.000 (60.6, 75.0)	0.472
SDP (mmHg)	136.10 ± 21.99	134.87 ± 17.43	131.00 ± 18.45	134.31 ± 18.88	0.58
DBP (mmHg)	77.97 ± 9.92	80.70 ± 10.88	78.48 ± 11.38	77.99 ± 10.12	0.329
HR (time/min)	76.000 (67.5, 86.0)	78.500 (70.8, 86.3)	75.000 (70.5, 88.0)	77.000 (68.8, 88.0)	0.747
Patient chief complaint					
Chest stuffiness	54 (19.15)	67 (23.76)	53 (18.79)	71 (25.18)	0.001**
Chest pain	1 (0.35)	2 (0.71)	16 (5.67)	37 (13.12)	0.194
Palpitation	1 (0.35)	2 (0.71)	6 (2.13)	13 (4.61)	0.137
Reexamination after PCI	6 (2.13)	1 (0.35)	2 (0.71)	3 (1.06)	0.069
Anamnesis					
Hypertension	42 (14.89)	15 (5.32)	55 (19.5)	46 (16.31)	0.019*
Diabetes	11 (3.9)	6 (2.13)	21 (7.45)	33 (11.7)	0.059
CHD	17 (6.03)	25 (8.87)	32 (11.35)	57 (20.21)	0.032*
Cerebral infarction	2 (0.71)	4 (1.42)	9 (3.19)	17 (6.03)	0.096
Vessel Lesion count					
Single vessel	8 (2.84)	38 (13.48)	19 (6.74)	8 (2.84)	0.082
Two-vessel	4 (1.42)	8 (2.84)	17 (6.03)	9 (3.19)	0.040*
Three-vessel	4 (1.42)	14 (4.96)	26 (9.22)	45 (15.96)	0.086
Four-vessel	1 (0.35)	2 (0.71)	3 (1.06)	11 (3.9)	0.160
Lesioned Vessel					
Left main branch	1 (0.01)	4 (1.42)	4 (1.42)	14 (4.96)	0.136
Left anterior descending artery	9 (0.35)	51 (18.09)	60 (21.28)	78 (27.66)	0.043*
Left circumflex artery	3 (3.19)	24 (8.51)	39 (13.83)	67 (23.76)	0.090
Right coronary artery branch	5(1.06)	31(10.99)	35(12.41)	67(23.76)	0.073

* *P *< 0.05.

** *P *< 0.01.

### 3.2 Tongue image features of classified patients

**Figure 4:**
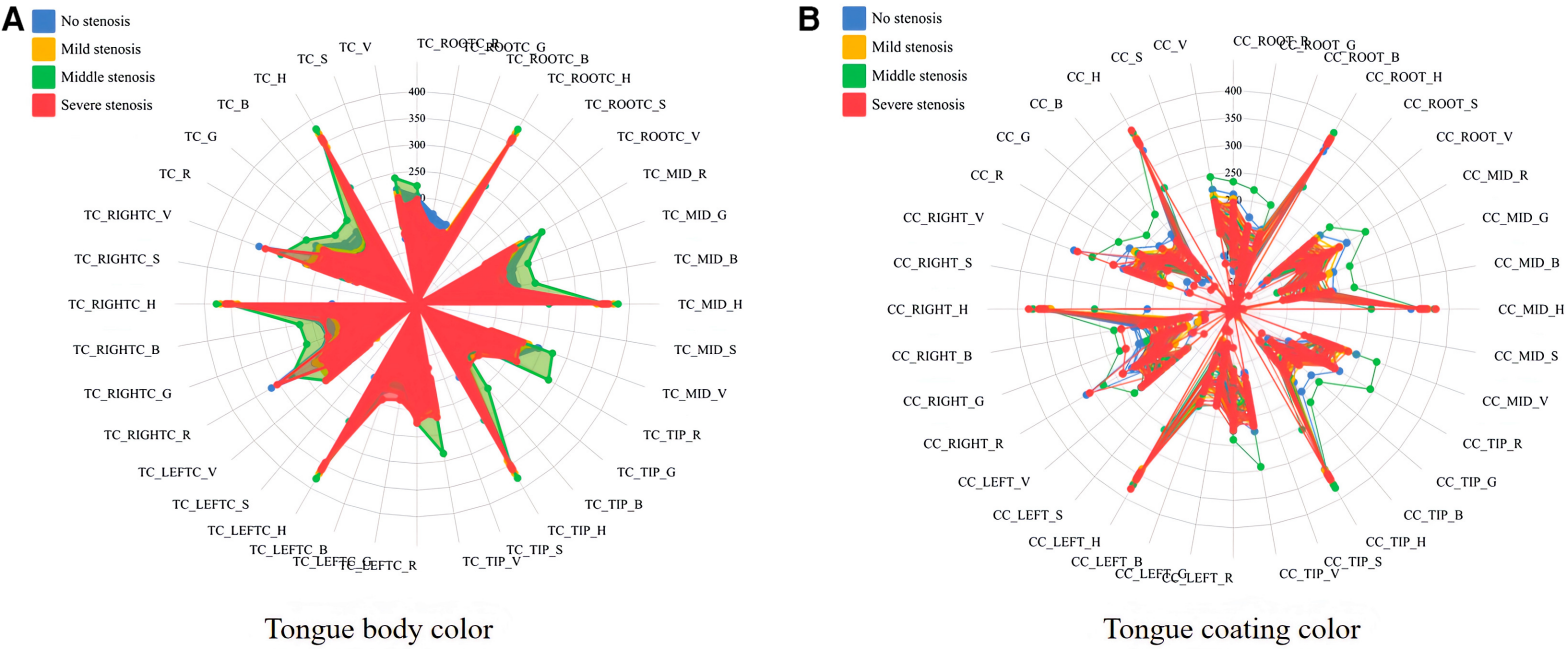
Radar map of tongue images with different degree of stenosis in patients with coronary angiography. **(A, B)** separately showed the inter-group differences in RGB and HSV model parameters of tongue body color (TC_) and coating color (CC_) in different degrees of coronary artery stenosis.

Based on the results shown in Table 2 and Figure 4, significant differences were observed in the overall tongue body, especially in TC_R (*P *= 0.000), TC_G (*P *= 0.003), TC_H (*P *= 0.001) and TC_S (*P *= 0.024). Notable distinctions were also identified in the overall tongue coating, especially in CC_R (*P *= 0.006), CC_B (*P *= 0.023), and CC_S (*P *= 0.001). Both RGB model and HSV model parameters exhibited significant variances between TC_MID_R, TC_MID_G, TC_MID_G and overall tongue coating.

**Table 2:** The difference of tongue color and tongue coating in different degree of coronary artery stenosis.

	Classification of stenosis *M* (P25, P75)				*P*
	No stenosis (*n* = 69)	Mild stenosis (*n* = 70)	Moderate stenosis (*n* = 65)	Severe stenosis (*n* = 78)	
TC_ROOT_R	126.258 (116.8, 146.5)	136.344 (110.0, 146.0)	124.921 (106.5, 123.8)	124.691 (116.0, 133.9)	0.007**
TC_ROOT_G	90.305 (82.2, 111.0)	89.299 (78.6, 104.2)	75.487 (69.9, 86.5)	89.478 (78.7, 98.3)	0.031*
TC_ROOT_B	92.560 (81.4, 110.5)	89.249 (76.8, 101.5)	75.081 (70.4, 84.3)	86.274 (70.8, 97.1)	0.011**
TC_ROOT_H	4.229 (1.2, 177.6)	3.146 (2.1, 91.0)	4.304 (1.0, 356.8)	4.928 (4.7, 5.8)	0.002**
TC_ROOT_S	0.292 (0.2, 0.3)	0.310 (0.3, 0.3)	0.343 (0.3, 0.4)	0.325 (0.3, 0.4)	0.021*
TC_ROOT_V	0.495 (0.5, 0.6)	0.538 (0.4, 0.6)	0.436 (0.4, 0.5)	0.495 (0.5, 0.5)	0.134
TC_MID_R	183.707 (175.7, 214.9)	191.295 (185.4, 215.2)	181.346 (166.5, 187.0)	192.381 (181.1, 203.1)	0.006**
TC_MID_G	138.523 (124.6, 153.5)	135.481 (124.7, 153.8)	119.863 (110.3, 141.8)	138.107 (127.4, 144.1)	0.013*
TC_MID_B	139.878 (123.9, 155.3)	142.120 (124.1, 158.9)	119.357 (104.0, 147.5)	140.719 (126.7, 144.6)	0.010*
TC_MID_H	2.075 (0.8, 357.8)	351.369 (1.1, 359.3)	348.332 (4.2, 358.7)	350.211 (1.1, 358.2)	0.122
TC_MID_S	0.281 (0.2, 0.3)	0.293 (0.3, 0.3)	0.341 (0.2, 0.4)	0.288 (0.3, 0.4)	0.045*
TC_MID_V	183.707 (175.7, 214.9)	191.295 (185.4, 215.2)	181.346 (166.5, 187.0)	192.381 (181.1, 203.1)	0.276
TC_TIP_R	185.345 (176.7, 202.5)	185.618 (175.7, 191.7)	173.590 (162.1, 190.6)	184.372 (167.6, 191.9)	0.001**
TC_TIP_G	130.158 (114.6, 131.7)	113.961 (108.0, 132.4)	112.879 (104.0, 117.7)	111.940 (101.1, 119.4)	0.001**
TC_TIP_B	132.148 (112.6, 136.7)	121.784 (108.1, 136.5)	114.442 (110.5, 119.9)	115.506 (105.1, 122.8)	0.000**
TC_TIP_H	357.111 (352.1, 358.4)	356.705 (349.7, 358.0)	354.948 (3.9, 357.9)	355.576 (351.6, 357.3)	0.002**
TC_TIP_S	0.333 (0.3, 0.4)	0.360 (0.3, 0.4)	0.359 (0.3, 0.4)	0.384 (0.3, 0.4)	0.000**
TC_TIP_V	185.345 (176.7, 202.5)	185.618 (175.7, 191.7)	173.590 (162.1, 190.6)	184.372 (167.6, 191.9)	0.001**
TC_LEFT_R	155.127 (143.3, 180.7)	184.474 (144.2, 211.1)	168.980 (159.2, 193.1)	177.716 (153.6, 201.9)	0.000**
TC_LEFT_G	103.468 (90.4, 125.5)	117.423 (81.9, 133.9)	114.730 (89.6, 124.5)	105.294 (99.3, 132.4)	0.087
TC_LEFT_B	101.563 (92.2, 123.4)	123.960 (86.9, 129.9)	118.868 (84.4, 128.8)	104.622 (98.9, 129.3)	0.048*
TC_LEFT_H	5.429 (1.7, 359.1)	356.689 (350.4, 357.9)	356.376 (7.0, 358.8)	344.184 (3.3, 358.2)	0.006**
TC_LEFT_S	0.326 (0.3, 0.4)	0.372 (0.3, 0.4)	0.355 (0.3, 0.4)	0.358 (0.3, 0.4)	0.268
TC_LEFT_V	155.127 (143.3, 180.7)	184.474 (144.2, 211.1)	168.980 (159.2, 193.1)	177.716 (153.6, 201.9)	0.000**
TC_RIGHT_R	184.857 (160.4, 216.0)	175.108 (164.3, 184.4)	165.743 (163.1, 185.9)	182.274 (171.4, 197.5)	0.003**
TC_RIGHT_G	125.877 (112.9, 159.0)	116.094 (106.7, 132.8)	115.165 (112.6, 124.7)	126.659 (113.5, 133.0)	0.001**
TC_RIGHT_B	133.239 (113.2, 160.9)	118.463 (100.4, 135.8)	114.748 (108.9, 125.3)	125.674 (114.4, 136.4)	0.002**
TC_RIGHT_H	350.365 (2.3, 358.9)	347.615 (2.0, 356.2)	5.015 (0.2, 357.4)	354.236 (1.5 3 58.5)	0.001**
TC_RIGHT_S	0.278 (0.2, 0.3)	0.315 (0.3, 0.4)	0.307 (0.3, 0.4)	0.341 (0.3, 0.4)	0.001**
TC_RIGHT_V	184.857 (160.4, 216.0)	175.108 (164.3, 184.4)	165.743 (163.1, 185.9)	182.274 (171.4, 197.5)	0.003**
TC_R	173.374 (164.3, 187.8)	171.922 (164.2, 197.0)	159.365 (154.1, 174.6)	180.906 (170.2, 185.8)	0.000**
TC_G	119.658 (111.2, 131.8)	124.526 (105.1, 132.9)	109.201 (102.0, 118.1)	127.309 (114.0, 127.5)	0.003**
TC_B	118.756 (110.3, 132.3)	124.371 (105.1, 141.1)	109.324 (104.1, 120.7)	127.021 (114.1, 128.2)	0.032*
TC_H	350.242 (1.0, 359.0)	355.701 (255.0, 359.6)	357.656 (6.9, 359.7)	352.912 (0.6, 358.4)	0.001**
TC_S	0.300 (0.2, 0.3)	0.325 (0.3, 0.4)	0.338 (0.3, 0.4)	0.314 (0.3, 0.4)	0.024*
TC_V	173.374 (164.3, 187.8)	171.922 (164.2, 197.0)	159.365 (154.1, 174.6)	180.906 (170.2, 185.8)	0.065
CC_ROOT_R	138.099 (113.3, 186.5)	137.601 (130.5, 179.1)	114.558 (91.4, 158.8)	147.688 (120.8, 158.1)	0.000**
CC_ROOT_G	95.034 (82.9, 149.6)	113.682 (95.9, 140.1)	87.873 (64.8, 105.2)	109.305 (95.8, 126.0)	0.000**
CC_ROOT_B	88.744 (79.6, 143.0)	102.449 (94.4, 133.2)	75.711 (61.8, 100.0)	100.126 (84.2, 125.5)	0.003**
CC_ROOT_H	17.754 (9.1, 355.1)	19.361 (11.4, 351.7)	14.549 (6.8, 352.1)	15.411 (14.7, 110.7)	0.146
CC_ROOT_S	0.272 (0.2, 0.3)	0.279 (0.2, 0.3)	0.339 (0.3, 0.4)	0.304 (0.2, 0.4)	0.012*
CC_ROOT_V	138.099 (113.3, 186.5)	137.601 (130.5, 179.1)	114.558 (91.4, 158.8)	147.688 (120.8, 158.1)	0.108
CC_MID_R	190.216 (181.5, 205.0)	195.914 (182.6, 206.8)	177.389 (170.6, 190.6)	190.289 (181.6, 199.7)	0.000**
CC_MID_G	147.081 (140.2, 154.7)	148.796 (130.7, 161.4)	138.336 (117.6, 143.3)	145.474 (135.9, 155.8)	0.006**
CC_MID_B	141.736 (135.7, 168.1)	146.356 (127.9, 167.2)	131.340 (103.6, 141.3)	139.086 (121.0, 150.7)	0.001**
CC_MID_H	329.048 (5.3, 355.2)	19.241 (8.7, 352.3)	12.830 (5.2, 352.2)	16.130 (11.1, 346.8)	0.194
CC_MID_S	0.252 (0.2, 0.3)	0.262 (0.2, 0.3)	0.311 (0.2, 0.4)	0.285 (0.2, 0.3)	0.001**
CC_MID_V	190.216 (181.5, 205.0)	195.914 (182.6, 206.8)	177.389 (170.6, 190.6)	190.289 (181.6, 199.7)	0.000**
CC_TIP_R	187.531 (171.4, 197.7)	191.923 (167.3, 200.3)	188.825 (157.3, 197.8)	191.762 (179.1, 197.8)	0.433
CC_TIP_G	133.741 (109.2, 144.7)	126.431 (110.0, 141.5)	125.153 (102.1, 135.5)	130.074 (111.3, 135.3)	0.262
CC_TIP_B	137.499 (110.2, 144.7)	128.444 (111.1, 148.6)	137.549 (98.7, 141.9)	137.197 (112.3, 140.3)	0.738
CC_TIP_H	352.020 (9.2, 358.0)	350.180 (16.8, 354.2)	351.205 (8.5, 354.6)	351.280 (13.2, 354.0)	0.274
CC_TIP_S	0.304 (0.3, 0.3)	0.322 (0.3, 0.4)	0.338 (0.3, 0.4)	0.327 (0.3, 0.4)	0.001**
CC_TIP_V	187.531 (171.4, 197.7)	191.923 (167.3, 200.3)	188.825 (157.3, 197.8)	191.762 (179.1, 197.8)	0.433
CC_LEFT_R	173.613 (156.9, 177.7)	197.320 (167.6, 204.8)	187.941 (151.2, 193.5)	187.234 (131.4, 203.2)	0.000**
CC_LEFT_G	119.744 (103.5, 137.7)	136.156 (110.4, 146.5)	132.705 (103.7, 141.5)	120.475 (93.3, 148.9)	0.141
CC_LEFT_B	112.230 (95.6, 138.1)	125.305 (104.6, 157.6)	137.354 (90.0, 147.8)	124.727 (76.9, 144.5)	0.037*
CC_LEFT_H	8.676 (2.0, 352.2)	351.397 (14.0, 355.3)	346.405 (7.6, 351.4)	12.574 (7.2, 349.1)	0.012*
CC_LEFT_S	0.299 (0.2, 0.4)	0.329 (0.3, 0.4)	0.307 (0.3, 0.4)	0.314 (0.3, 0.4)	0.144
CC_LEFT_V	173.613 (156.9, 177.7)	197.320 (167.6, 204.8)	187.941 (151.2, 193.5)	187.234 (131.4, 203.2)	0.109
CC_RIGHT_R	189.707 (164.3, 214.9)	178.031 (165.1, 199.0)	170.701 (153.0, 192.5)	187.409 (174.9, 200.3)	0.003**
CC_RIGHT_G	141.293 (112.2, 158.9)	135.021 (111.6, 146.6)	122.715 (110.1, 147.1)	140.329 (126.2, 145.2)	0.002**
CC_RIGHT_B	140.755 (114.4, 174.5)	123.084 (98.9, 142.7)	121.215 (112.8, 147.2)	131.119 (115.6, 143.1)	0.006**
CC_RIGHT_H	342.315 (8.4, 355.8)	347.575 (13.0, 354.1)	13.053 (5.6, 351.7)	12.639 (10.5, 349.1)	0.003**
CC_RIGHT_S	0.283 (0.2, 0.3)	0.293 (0.3, 0.4)	0.308 (0.2, 0.3)	0.295 (0.2, 0.4)	0.005**
CC_RIGHT_V	189.707 (164.3, 214.9)	178.031 (165.1, 199.0)	170.701 (153.0, 192.5)	187.409 (174.9, 200.3)	0.611
CC_R	177.943 (166.9, 194.7)	181.921 (162.9, 201.8)	168.020 (154.6, 181.3)	181.207 (168.5, 189.7)	0.006**
CC_G	127.438 (118.3, 148.9)	143.701 (112.6, 150.2)	120.820 (102.1, 132.2)	135.557 (127.5, 145.5)	0.11
CC_B	130.476 (117.0, 153.3)	132.273 (115.8, 149.0)	115.998 (109.3, 130.3)	135.903 (115.3, 137.9)	0.023*
CC_H	11.791(5.6, 356.8)	18.480(7.9, 353.5)	13.235(5.6, 351.8)	13.917(7.1, 347.2)	0.265
CC_S	0.260(0.2, 0.3)	0.275(0.3, 0.3)	0.310(0.3, 0.3)	0.290(0.2, 0.3)	0.000**
CC_V	177.943(166.9, 194.7)	181.921(162.9, 201.8)	168.020(154.6, 181.3)	181.207(168.5, 189.9)	0.072

TC, represents the tongue color; CC, represents the tongue coating color; ROOT, LEFT, RIGHT, MID, TIP, represent different parts of the tongue respectively.

* *P *< 0.05.

** *P *< 0.01.

### 3.3 Model training and validation

**Figure 5:**
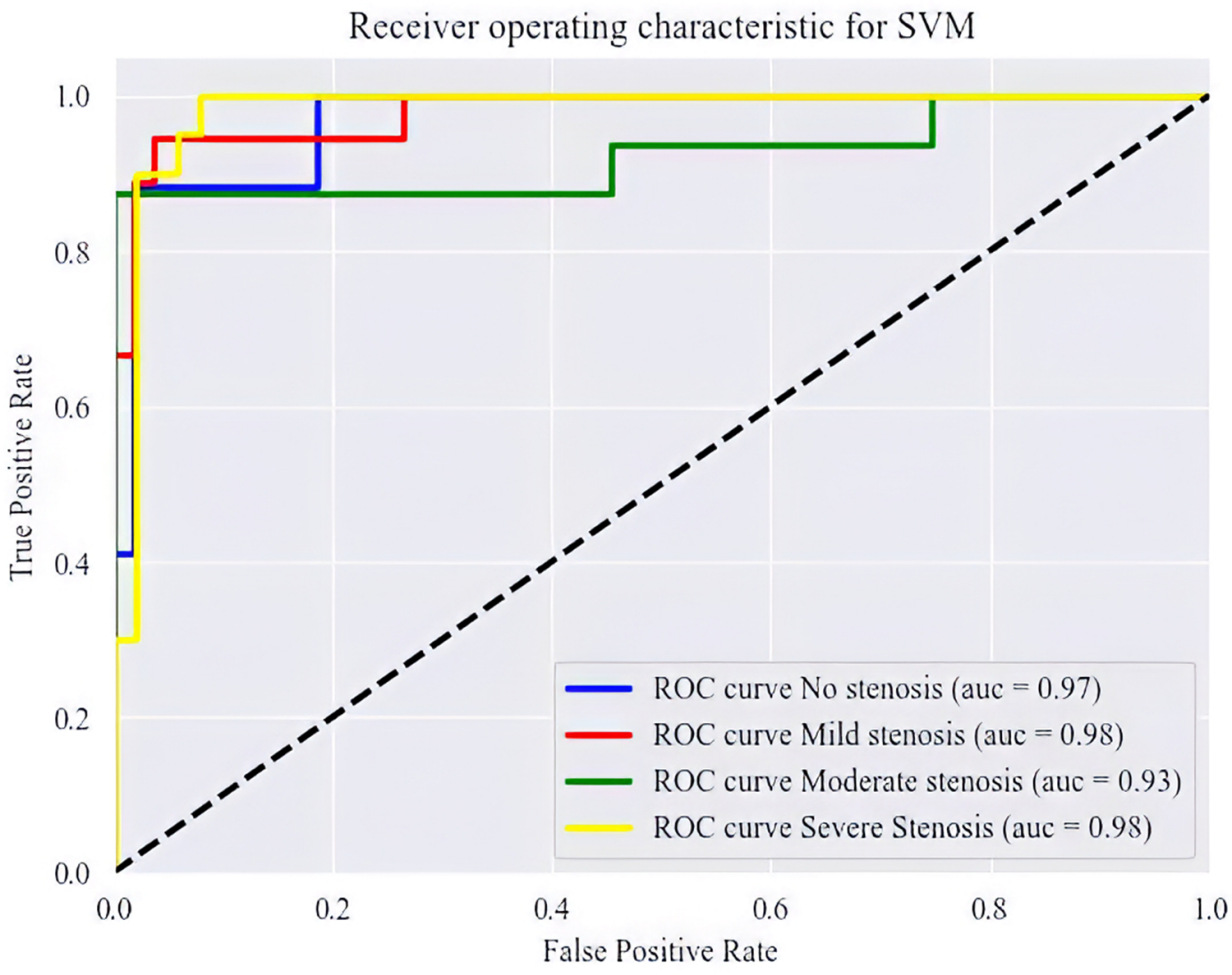
The ROC curve in different groups.

The SVM, LR, RF, and XGBoost models were employed for classifying coronary artery stenosis to train and validate the tongue color parameters of patients with varying degrees of stenosis. As depicted in Table 3, all four algorithms exhibited high verification results. In terms of the training set, SVM and RF demonstrated superior prediction performance with accuracy, precision, recall, and F1 scores exceeding 90%. For the test set, SVM and XGBoost displayed the highest prediction performance. We integrated the training process and hierarchical 10-fold cross-validation for each model using the training dataset. The visualization of each fold's results from the merged dataset can be found in the Supplementary Material. Based on evaluation results from both sets (training and validation), SVM proved to be highly stable based on tongue color parameters while also being the optimal predictive model for determining coronary artery stenosis severity. Figure 5 illustrates a ROC curve showcasing SVM's performance using tongue color parameter data. Different colored lines represent various degrees of coronary artery stenosis within this ROC curve plot. Results indicate that SVM exhibits a predictive ability greater than 0.9 across different groups; furthermore, it excels at identifying mild and severe stenoses (AUC = 0.98).

**Table 3:** Results on the classification of machine learning model.

Model	Training results (mean ± SD)				Validation results (mean ± SD)			
	Accuracy	Precision	Recall	F1 Score	Accuracy	Precision	Recall	F1 Score
SVM	0.9102 ± 0.0614	0.9280 ± 0.0544	0.9102 ± 0.0614	0.9093 ± 0.0621	0.9014	0.9008	0.9014	0.9
LR	0.8108 ± 0.0620	0.8365 ± 0.0664	0.8108 ± 0.0620	0.8004 ± 0.0654	0.7606	0.7618	0.7606	0.7603
RF	0.9147 ± 0.0555	0.9269 ± 0.0504	0.9147 ± 0.0555	0.9135 ± 0.0567	0.8873	0.8888	0.8873	0.8869
XGBoost	0.8861 ± 0.0573	0.8948 ± 0.0543	0.8861 ± 0.0573	0.8844 ± 0.0580	0.9014	0.9049	0.9014	0.9019

SVM, support vector machine; LR: logistic regression; RF, random forest; SD, standard deviation; XGBoost, extreme gradient boosting.

### 3.4 SHAP value of features

**Figure 6:**
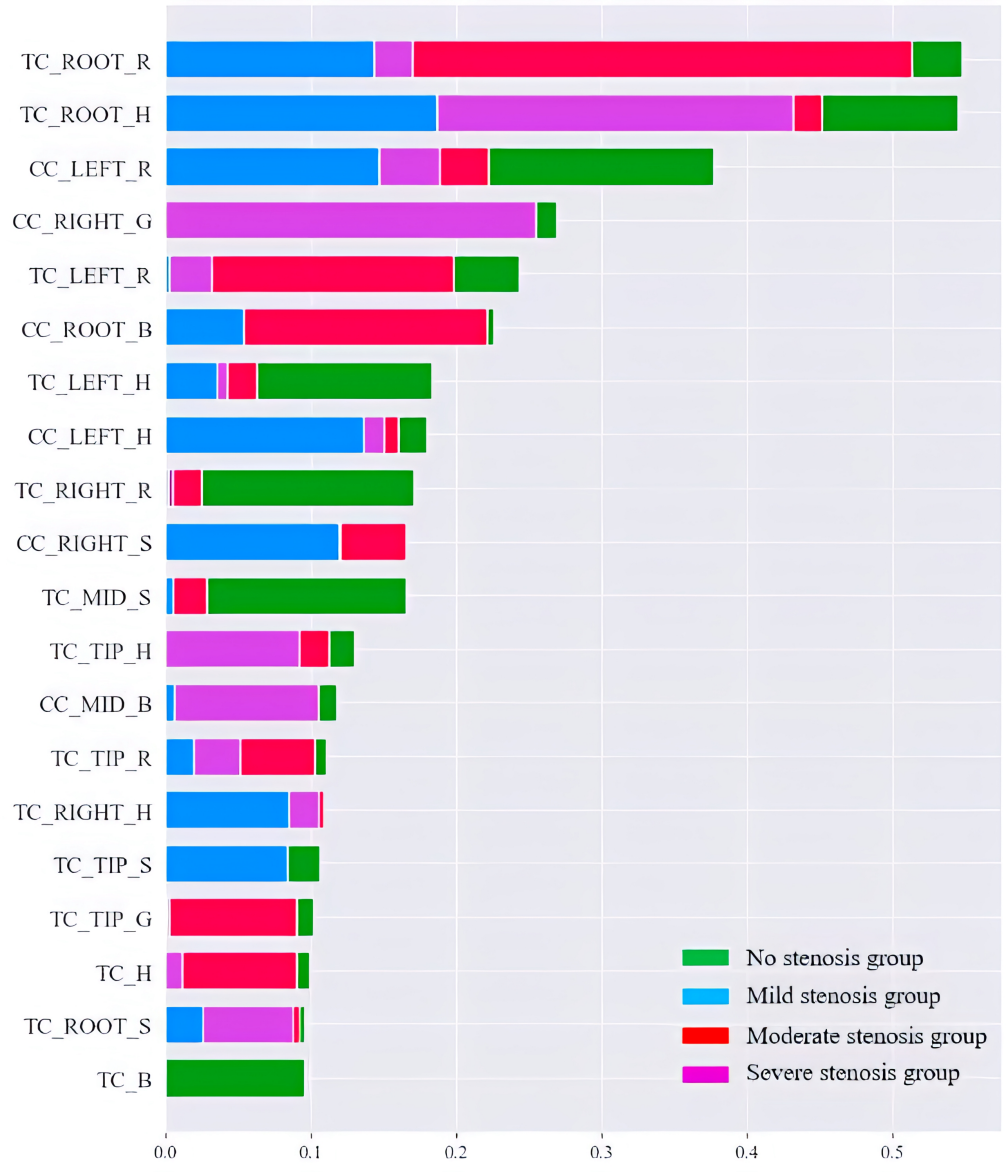
Summary plot of tongue color features for coronary artery stenosis.

Based on the SVM model visualization report, the top 20 tongue color features with the most output contribution are shown in Figure 6, which are: TC_ROOT_R; TC_ROOT_H; CC_LEFT_R; CC_RIGHT_G; TC_LEFT_R; CC_ROOT_B; TC_LEFT_H; CC_LEFT_H; TC_RIGHT_R; CC_RIGHT_S; TC_MID_S; TC_TIP_H; CC_MID_B; TC_TIP_R; TC_RIGHT_H; TC_TIP_S; TC_TIP_G; TC_H; TC_ROOT_S and TC_B. These characteristics are highly correlated with the degree of vascular stenosis. TC_ROOT_R; TC_LEFT_R and CC_ROOT_B are more important for predicting moderate stenosis than other stenosis degrees.

**Figure 7:**
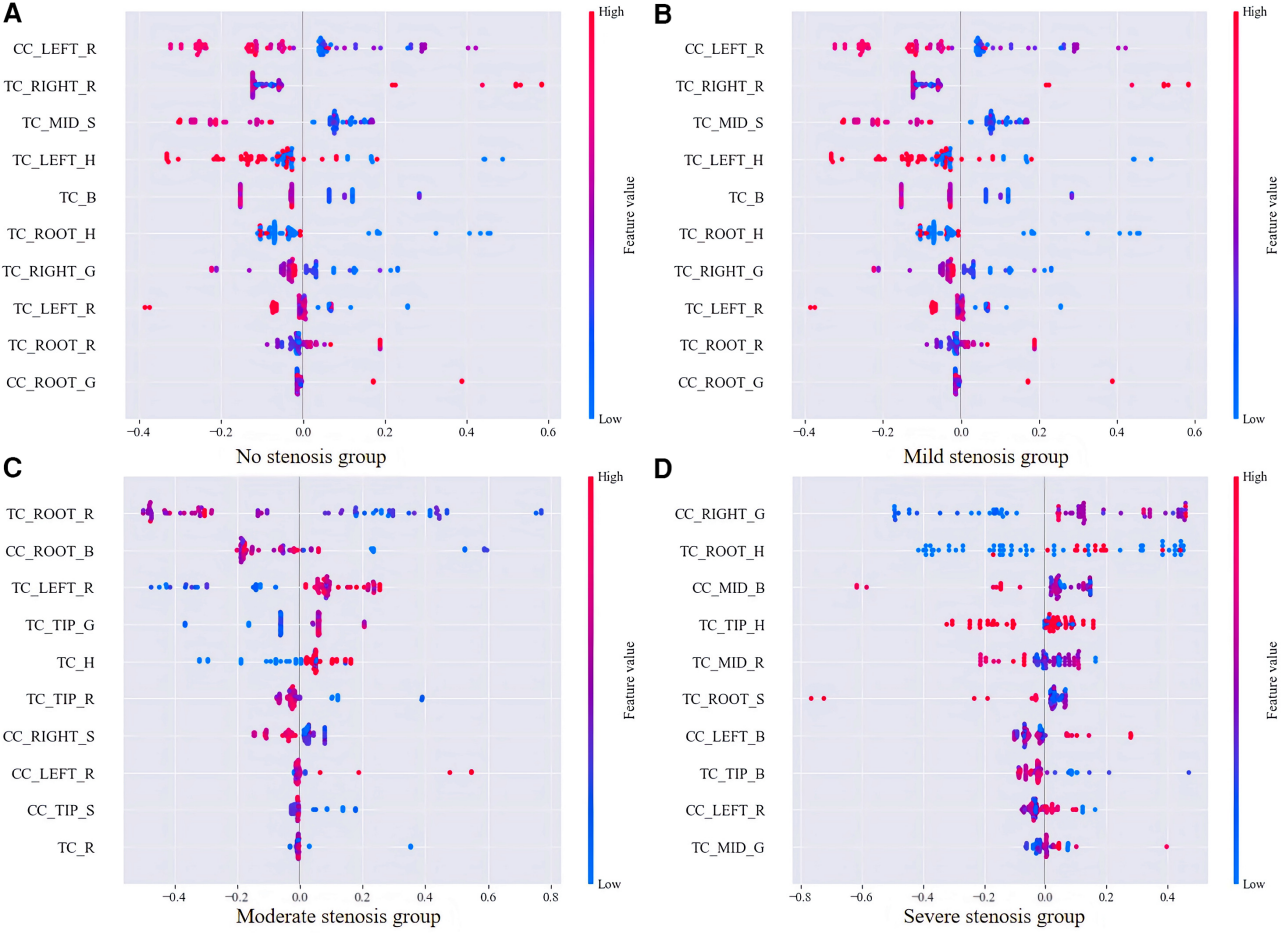
SHAP value plot for SVM which contains top 10 features of four stenosis groups. **(A–D)** charts correspondingly displayed the influence of the most predictive tongue body and tongue coating color parameters on different degrees of coronary artery stenosis.

In order to visualize the output contribution of tongue color features of different groups more intuitively, four cellular graphs of SHAP value further showed the influence of tongue color parameter values on different degrees of coronary stenosis. Each row represented a tongue color feature and each dot represented a topic. The color represented the size of the feature parameter, and the higher the value was, the red color was. The results were shown in Figure 7: high values of TC_RIGHT_R, TC_ROOT_R, and CC_ROOT_G, and low values of CC_LEFT_R, TC_MID_S, TC_B, and TC_ROOT_H could positively predict no coronary stenosis; high values of CC_LEFT_R, TC_ROOT_R, CC_LEFT_H, CC_RIGHT_S, CC_ROOT_B, CC_ROOT_G, and TC_S, and low values of TC_ROOT_H and TC_TIP_S could positively predict mild coronary stenosis; high values of TC_LEFT_R, TC_H, and CC_LEFT_R, and low values of TC_ROOT_R, TC_TIP_R, CC_TIP_S, and TC_R could positively predict moderate coronary stenosis; high values of CC_RIGHT_G, CC_LEFT_B, and low values of TC_TIP_B, and TC_ROOT_S could positively predict severe coronary stenosis.

## 4 Discussion

This study conducted a retrospective cohort analysis of tongue images from 282 patients who underwent coronary angiography, revealing significant variations in the RGB and HSV parameters of tongue texture and coating among individuals with different degrees of coronary artery stenosis. These findings offer preliminary evidence supporting the potential application of tongue color analysis for diagnosing coronary artery disease. Furthermore, employing four classical prediction models based on tongue color parameters, this study demonstrated that the SVM model exhibited the highest AUC value, indicating superior predictive capability. Collectively, these results suggest that tongue color analysis holds practical value as an adjunctive diagnostic tool for coronary heart disease.

### 4.1 Relationship between tongue image and degree of coronary stenosis

Tongue diagnosis is one of the unique diagnostic methods of TCM. The findings of relative TCM research have indicated that changes in the appearance of the tongue can serve as objective indicators for variations in human Qi and blood, the nature of pathogenic factors, the location and progression of diseases, as well as their prognoses (5, 9). Coronary heart disease (CHD) primarily arises from atherosclerosis within coronary arteries leading to vessel narrowing or blockage, thereby impairing blood supply to the heart (13). Clinical observations revealed that patients often experience symptoms such as palpitations, chest tightness, chest pain, and shortness of breath which are associated with impaired cardiac function or circulatory system disorders (28). Studies have shown a certain correlation between changes in tongue appearance and the degree/severity of coronary artery disease (5, 9). For instance, Duan et al. conducted a cross-sectional study investigating the relationship between alterations in tongue appearance and CHD severity; they found positive correlations between characteristics like tongue color/shape with CHD severity suggesting that parameters related to tongue appearance could be utilized for predicting CHD severity among patients diagnosed with this condition (29). In addition, in cases of severe coronary artery obstruction, the patient's tongue tends to be darker in color, accompanied by a thicker greasy coating (9). These changes reflect the pathological status of poor Qi-blood circulation and accumulation of phlegm and dampness in the patient. It provides a valuable reference for evaluating the condition of coronary heart disease (3).

In this study, we observed that the root and right part of the tongue of patients in the stenosis-free group had high R values, with a reddish color, and the S value saturation in the middle part of the tongue was higher, with a darker color. The R values and overall saturation of the tongue of patients with mild stenosis were similar to those of patients with stenosis-free, but the color of the tongue coating was different, with the left and right parts of the tongue coating being reddish, and the G and B values of the tongue coating root were high, with a blue-green color. The tongue color of the tongue and the left part of the tongue coating of patients with moderate coronary stenosis were reddish, the tongue root and tip of the tongue coating were blue-green, and the overall color of the tongue was lighter than that of patients with stenosis-free and mild stenosis. The tongue tip of patients with severe coronary stenosis was reddish, and the left and right sides of the tongue coating were blue-green. Our results are similar to other clinical results. The tongue images of patients in the no-stenosis group showed a ruddy tongue with dark or blue-green tongue coating, which may be related to their state of fullness of Qi and blood and good circulation (4, 9). The tongue images of patients with mild stenosis were similar to those of patients in the stenosis-free group, but the left and right parts of the tongue coating were reddish, which may imply the existence of local Qi and blood stasis or heat images. This tongue image may mean that the cardiovascular state of these patients is relatively good, and although some of them have mild coronary stenosis, it has no significant effect on the overall Qi and blood circulation. With the deepening of the degree of stenosis, the tongue color of patients with moderate stenosis begins to become lighter, and the tongue root and tip appear blue-green, which may be related to local blood circulation disorder or metabolic abnormality. When it comes to the severe stenosis stage, the tongue tip of patients is obviously red, and the tongue coating on both sides also appears blue-green, which may be related to hyperactivity of heart fire or more serious local metabolic abnormality.

Studies have demonstrated that cardiac hyperactivity may result in an elevated metabolic rate, thereby potentially increasing the burden on the heart and impacting its normal function (30). Similar to existing hemodynamic research, mild stenosis typically exerts minimal influence on hemodynamics, while moderate and severe stenosis can significantly reduce blood flow velocity and coronary artery perfusion. Research has indicated that severe coronary stenosis is often attributed to the accumulation of excessive lipids, cholesterol, and other substances within the inner wall of the coronary artery, leading to atherosclerotic plaque formation (31). These plaques progressively expand and encroach upon vessel lumen space, ultimately restricting coronary blood flow and giving rise to symptoms such as myocardial ischemia and angina pectoris (32). Future investigations could integrate physiological and biochemical indicators from clinical practice to further elucidate the specific mechanisms underlying the relationship between the degree of coronary artery stenosis and tongue color parameters. This would enhance our understanding of the etiology and progression of coronary artery disease while providing more effective means for clinical diagnosis and treatment.

### 4.2 Application of machine learning to predict the degree of coronary stenosis

Although there was limited research on machine learning in tongue detection of coronary artery stenosis, the application of machine learning in tongue detection of coronary artery stenosis has great potential and significance (7, 9, 33, 34). Coronary angiography is the gold standard for the diagnosis of coronary artery stenosis, but it is costly, complex and has certain risks. As an important diagnostic method of TCM, tongue detection has the advantages of simplicity, non-invasiveness and intuition. Through machine learning technology, we can deeply mine and analyze the features of tongue images and establish a model to predict coronary artery stenosis, thus providing an auxiliary basis for the decision-making of coronary angiography. The four classifiers used in this study, namely SVM, LR, RF and XGBoost, all showed high prediction performance in the training and validation process. Detailed work was carried out in data preprocessing and feature selection, which effectively improved the prediction performance of the model. Among them, the SVM model showed the best prediction effect due to its strong classification ability and generalization performance. This may be related to the advantages of the SVM model in dealing with high-dimensional data, solving small sample learning problems and dealing with nonlinear problems (35). However, it is worth noting that the accuracy and reliability of tongue color parameters are affected by a variety of factors, such as doctors' experience, observation environment, etc. Therefore, when using tongue color parameters for clinical data analysis, a variety of factors need to be considered comprehensively to improve the accuracy and reliability of diagnosis.

### 4.3 Advantages and limitations

Although this study implemented rigorous quality control measures throughout the process of data collection, processing, and analysis to ensure the accuracy and reliability of the findings, as well as systematically investigated the association between tongue color and coronary artery stenosis, there are still certain limitations. Firstly, the sample size is relatively small, which may not fully capture the true scenario. Future studies will expand the sample size, and external verification will be carried out to better verify the persuasiveness of this prediction model. Secondly, this study primarily focused on examining the relationship between tongue color parameters and the severity of coronary artery stenosis without considering other tongue characteristics such as coating morphology or texture. Subsequent research should comprehensively incorporate additional tongue features to improve prediction accuracy. Furthermore, being a retrospective study introduces potential information bias and selection bias. Prospective designs in future studies can mitigate these biases' impact.

## 5 Conclusion

This study explored the variation of tongue color characteristics in patients with different degrees of coronary artery stenosis through retrospective cohort analysis, and verified the effectiveness of tongue color parameters in predicting the degree of coronary artery stenosis. These findings not only provide a modern scientific basis for traditional Chinese medicine tongue diagnosis, but also provide a new idea and method for the early diagnosis and condition assessment of coronary heart disease. Future research can further expand the scope of tongue features, optimize the prediction model and explore its application value in other cardiovascular diseases.

## Data Availability

The raw data supporting the conclusions of this article will be made available by the authors, without undue reservation.
