# Supplementary material for: Tongue color parameters in predicting the degree of coronary stenosis: a retrospective cohort study of 282 patients with coronary angiography
Source: Front Cardiovasc Med. 2024 Aug 30;11:1436278. doi: 10.3389/fcvm.2024.1436278 (PMC11392741; doi:10.3389/fcvm.2024.1436278)
Supplement: Supplementary file 1 [file Datasheet1.pdf]

## Supplementary Material

### Classification metrics

The primary indicators employed to assess model performance are accuracy, F1 score, and AUC. A summary of the model's performance can be obtained with a confusion matrix. Within this matrix, true positives (TP) refer to the count of correctly predicted different conditions of coronary stenosis subjects, while true negatives (TN) represent the count of accurately predicted non-specific group of coronary stenosis. False negatives (FN) signify the count of non-specific group of coronary stenosis subjects incorrectly predicted, while false positives reflect the count of fits the specific coronary stenosis group subjects incorrectly predicted.

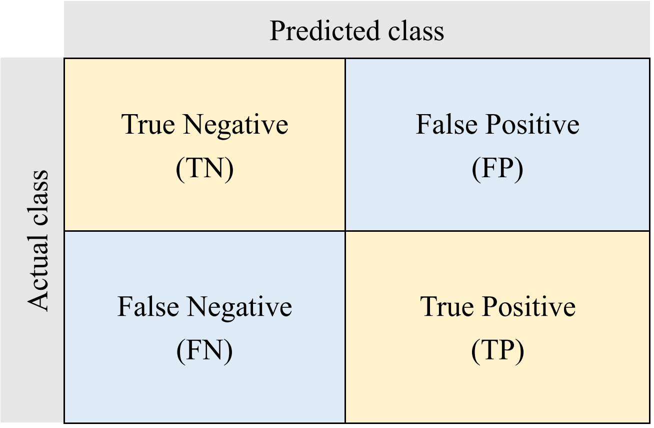


Supplementray Figure 1. The confusion matrix.

Accuracy is a widely employed metric for evaluating model performance in classification tasks. It is calculated as the ratio of correct predictions to the total number of observations. Accuracy provides a measure of the proportion of correct predictions made by the model.

$$Accuracy = \frac{TN+TP}{TN+FN+FP+TP} \quad \text{Eq. (A.1)}$$

Precision, also known as the positive predictive value, quantifies the accuracy of positive predictions made by a model. It is calculated as the ratio of true positives to the sum of all positive predictions. A high precision value indicates a lower false positive rate, implying that the model has a greater ability to correctly identify positive instances.

$$Precision = \frac{TP}{TP+FP} \quad \text{Eq. (A.2)}$$

Recall, also referred to as sensitivity, measures the proportion of true positives to the sum of true positives and false negatives. A higher recall value indicates a lower false negative rate, implying that the model is more successful in accurately detecting positive cases. In the context of the present study, a high recall signifies the model's effectiveness in correctly predicting the majority of the specific coronary stenosis group subjects.

$$Recall = \frac{TP}{TP+FN} \quad \text{Eq. (A.3)}$$

The F1 score assesses a model's precision and recall capabilities by calculating their harmonic mean. It provides a comprehensive evaluation of both precision and recall. The F1 score ranges from 0 (representing the worst performance) to 1 (indicating the best performance). By considering both precision and recall, the F1 score offers a balanced measure of the model's overall effectiveness.

$$F1\ score = 2 \times \frac{Precision \times Recall}{Precision + Recall} \quad \text{Eq. (A.4)}$$

The Area under the Receiver Operating Characteristic curve (AUC/ROC AUC) is a valuable metric for assessing and visualizing the classification ability of a model. The ROC graph provides insight into the relationship between the true positive rate (TPR) and the false positive rate (FPR). The AUC value ranges from 0 to 1.0, where a value of 0.5 represents random guessing, and a larger AUC value indicates a better-performing model. By analyzing the AUC, one can gain an understanding of the model's discriminative power and its ability to distinguish between different classes.

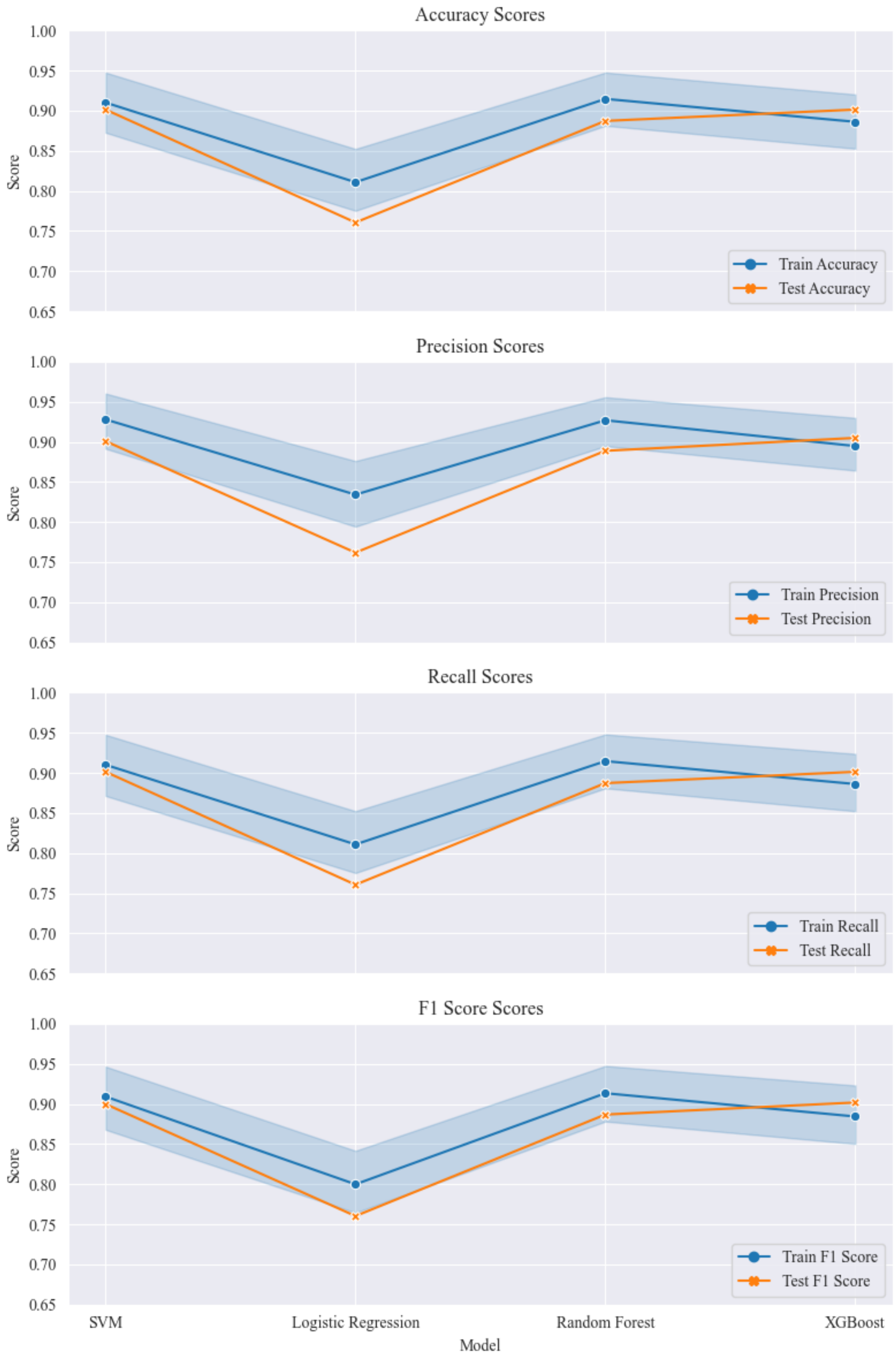


Supplementary Figure 2. Ten cross-validation graphs of training sets and validation sets of the four classifiers
